# Research capacity strengthening methods and meanings: negotiating power in a global health programme on violence against women

**DOI:** 10.1136/bmjgh-2024-015376

**Published:** 2024-12-11

**Authors:** Nerissa Tilouche, Beatriz Kalichman, Sandi Dheensa, Evelina Rossi, Claire Hawcroft, Ana Flavia d'Oliveira, Heba Owda, Loraine J Bacchus

**Affiliations:** 1 Faculty of Public Health and Policy, London School of Hygiene & Tropical Medicine, London, UK; 2 Faculty of Medicine, Sao Paulo University, Sao Paulo, Brazil; 3 Bristol Medical School, University of Bristol, Bristol, UK; 4 Faculty of Medicine and Health Sciences, An-Najah National University, Nablus, West Bank, State of Palestine

**Keywords:** Global Health, Qualitative study

## Abstract

**ABSTRACT:**

**Background:**

There has been much critical reflection among global health researchers about how power imbalances between high-income countries and low- and middle-income country collaborators are perpetuated through research programmes. Research capacity strengthening (RCS) is considered both a mechanism through which to redress structural power imbalances in global health research and a vehicle for their perpetuation. This paper examines the RCS programme of a multi-county study on violence against women, focussing on how it addressed power imbalances between countries and the challenges involved in doing so. It provides specific examples and lessons learnt.

**Methods:**

18 semi-structured interviews were conducted online with group members from all five countries involved in the collaboration between April and June 2020. Reflexive thematic analysis, with inductive and deductive approaches was adopted.

**Findings:**

Participants articulated their understandings of RCS as an opportunity for (1) mutual learning, understanding and collaboration and (2) personal and team career development. Participants perceived the RCS programme activities to simultaneously reinforce and challenge power asymmetries within global health research. Power dynamics within the RCS programme operated across three levels; the global health research environment, the research group level and within individual country teams. Participants described structural barriers at all three levels, but felt there were more opportunities to challenge power imbalances at the research group level.

**Conclusion:**

Despite a strong commitment to addressing power imbalances through the RCS programme, progress was often hampered by the fact that these inequalities reflected broader structural issues in global health, as seen within Healthcare Responding to Violence and Abuse. The programme sometimes faced tensions between enhancing researchers’ careers while building capacity under the current model and creating social value or challenging epistemic and normative structures. Participants clearly expressed concerns about power imbalances within the partnership and were keen to address them through the RCS programme. This led to a steep learning curve and significant adaptations within the RCS programme to navigate these issues within existing structural limitations.

**WHAT IS ALREADY KNOWN ON THIS TOPIC?**

**WHAT THIS STUDY ADDS**

- Unlike studies focused solely on research outputs, this study also explored the experiences and understandings of RCS, the potential for RCS to challenge inequity, and the challenges involved in doing so, providing concrete examples.
- This multi-country RCS research study was conducted outside the continent of Africa, where most existing RCS evidence originates.

**HOW THIS STUDY MIGHT AFFECT RESEARCH, PRACTICE OR POLICY**

- In offering concrete examples of challenges and solutions to issues of power dynamics in RCS programmes this study can help to guide future endeavours in the area.
- RCS is often an essential component of global health funding initiatives and is considered an important mechanism for challenging power dynamics in partnerships.
- This paper exemplifies ways in which the global health environment can hinder or potentialise these efforts.
- English language dominance in global health research is symptomatic of, and perpetuates, power imbalances between countries.
- Funders should consider incentivising publishing in local journals or international journals could be required to publish in different languages.

## Background

 Recent years have seen an increased recognition that global and contemporary health research inequities are rooted in political institutions and research environments established under colonialism, perpetuated through international politics and development.^1 2^ Global health researchers have resultantly begun to critically reflect on how research programmes, which often prioritise high-income country (HIC) research agendas and academic careers, reproduce power imbalances in the field.^2^,^4^ Researchers have interrogated research capacity strengthening (RCS) as both a contributor to this dynamic and a means to address it.^5 6^ Shiffman’s taxonomy defines power in global health as influencing others’ abilities to control their circumstances. It includes economic, epistemic and normative power, the last two respectively referring to the power derived from claims to expertise and moral authority.^7^ According to Shiffman, although economic power is often recognised and interrogated, the other two are less so, but considering the role of all three in shaping structures and production in global health is important.

A systematic review revealed that global health definitions in the literature disproportionately represent HIC interests and priorities^8^: the term ‘global health’ originates from HICs.^5^ The shift from ‘international’ to ‘global’ health moved governance, funding and research delivery away from a focus on between-state agreements to include private firms, philanthropic and non-governmental organisations, and public–private partnerships, some view this transition as linked to the increased permeation of neoliberal ideas in the field.^9 10^ Others criticise biomedical researchers’ and economists influence in it, as it restricts ownership and diversity of perspectives and reduces social and political issues to biomedical ones, thereby depoliticising them.^11^

These ideological and discursive powers may significantly shape global health systems’ direction and impact.^47 9 12^,^15^

In international collaborations with HIC grant holders, they often lead research agendas and strategies, giving them power.^16 17^ A consequence is a lack of training and research that addresses the needs in the Global South.^18^ Weber *et al*^19^ highlight that knowledge from low- and middle-income country (LMIC) partners is often undervalued, while theories and ethical frameworks ‘imposed’ by HIC researchers and institutions are afforded greater value.

Weber and colleagues touch on Shiffman’s (2014) idea of epistemic power by highlighting that HICs get to shape the research agenda, methodologies and knowledge production processes, thereby shaping the outcomes and impact of global health research. Abimbola raises a related issue^20^: he emphasises that the critical aspect of academic publishing is the audience and location of publication. During a webinar within HERA’s RCS programme, as a journal editor, he argues that dissemination focused on international publications negatively affects policy change by driving knowledge away from where it is produced, questioning current systems’ effectiveness for driving health policy change in LMICs.^21^

Power dynamics in global research on violence against women (VAW) present specific ethical challenges due to the risks to researchers and survivors, including (re-)traumatisation, risk to safety and exploitation. When unaddressed, power disparities within research teams and collaborations, and between researchers and participants, can exacerbate those risks.^19 22^ The global nature of this work also makes it important to take account of different contexts to guarantee that research and interventions are relevant and appropriate for addressing VAW drivers in distinct settings. However, these efforts can be hampered by power imbalances between those who formulate research and interventions and those who participate in them, disparities which are often related to global inequalities and colonial legacies.^23 24^

RCS can be defined as ‘any efforts to increase the ability of individuals and institutions to undertake high-quality research and to engage with the wider community of stakeholders’.^12^ Many funders now require an explicit RCS component in research projects.^25^ The literature presents RCS as both a tool to address power imbalances in partnerships, and, paradoxically, a mechanism that can reinforce or exacerbate them. For instance, its requirements in partnerships can imply an *assumed* lack of LMIC capacity: *reciprocal* RCS between HIC and LMIC is rarely acknowledged.^26^ Unidirectional RCS is often rooted in Eurocentric ideas about knowledge, its origins, and validity. These dismiss different forms of capacity, favouring a universalised idea of science, its uses and benefits, erasing contextual specificities and needs.^5^ This tendency is especially important to examine in global health research, since RCS can depoliticise inequalities, masking them as technical capacity gaps,^5^ much like the biomedicalisation of health issues does for global health.^14^ In other words, RCS can contribute to the idea that problems faced by LMICs are a matter of technical deficiencies rather than due to political and economic factors, thereby encouraging technocratic responses.^5^ Examining these issues in the VAWG research and intervention context is crucial, since there is mounting discontent in this field with HIC-LMIC power imbalance in funding and knowledge production and distribution, with the imposition of universalising ideas and methodologies, often originating from HICs.^23 27 28^ There is a risk that RCS interventions, focused on advancing careers within a research field moulded by HIC standards of excellence may compel LMIC researchers to reproduce those models, adopt an agenda that HIC funders consider relevant, and focus their publishing efforts in English language journals inaccessible to their national audience rather than prioritising knowledge and actions more relevant to their own context’ needs.^5 29^

Although the literature criticises RCS as often perpetuating power inequalities in partnerships, this perpetuation is not seen as an inherent flaw, but rather due to RCS models that fail to account for LMIC needs or strengths, instead perpetuating top-down approaches.^5 24 30^ Authors highlight that engaging different actors in setting RCS goals and ensuring the implementation enhances ownership for participants is crucial in ensuring that its outcomes advance capacity in ways that are useful and meaningful for all involved.^6 24^ There is, for instance, RCS in South-South partnerships focusing on recognising and building on what countries have to offer, instead of promoting a passive or unidirectional transfer of technology and knowledge.^30^ Focusing on broader RCS objectives Mormina and Istratii (2023) propose that RCS should focus on creating social value, understood as ‘positive impact to the equitable long-term wellbeing and resilience of individuals, communities and wider society’ (p4). This perspective is significant: it encourages reflection on the broader purpose of RCS and research in general.

Our study explored understandings of power within the RCS programme of the Healthcare Responding to Violence and Abuse (HERA) global health research group, which aimed to strengthen health system responses to VAW in LMICs through research and interventions. It also examined structural power dynamics operating across the programme and explored factors that possibly replicated or offered opportunities to redress power imbalances. Two UK universities, collaborating with academic partners in Brazil, Nepal, occupied Palestinian territories (oPt) and Sri Lanka co-led HERA.

**Figure 1:**
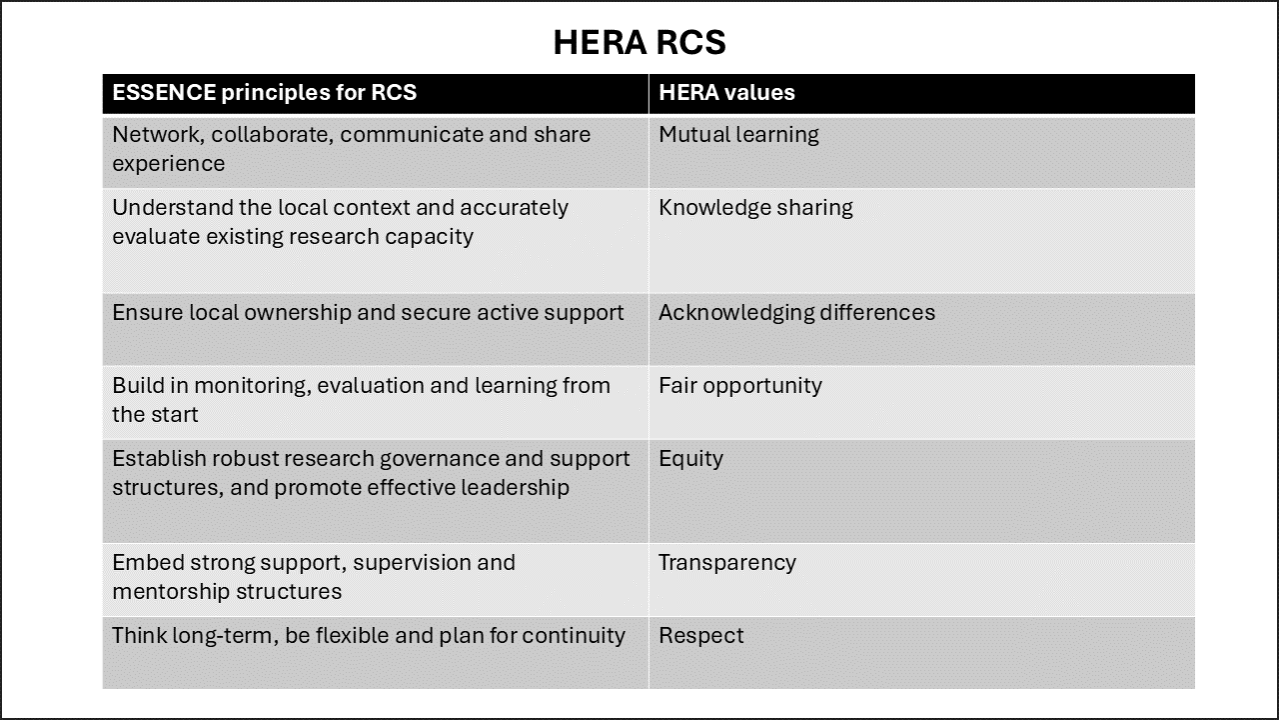
HERA RCS values and principles. HERA, Healthcare Responding to Violence and Abuse; RCS, research capacity strengthening.

The RCS programme, integral to HERA’s research agenda, focused on consensus-building around core values (figure 1), assessing development needs, setting agendas, and planning training activities and workshops. The agenda emphasised participatory planning and iterative monitoring. ESSENCE principles for RCS informed the programme^18^ (figure 1).

RCS was core to HERA since the project’s inception and was monitored with country teams, for example, in monthly reporting. The programme ran in 2018–2021 (end of the research), but continued informally, with researchers collaborating in different projects and sharing opportunities. It included activities organised by both the programme training leads, researchers officially appointed to coordinate RCS, and other researchers in different teams. Activities (table 1) ranged from formal events, for example, workshops, to informal initiatives, like mentoring and training sessions, making the programme diverse, decentralised and responsive to different teams’ needs. Early career researchers (ECRs), predominantly LMIC-based, were the primary focus, resulting in concentrated efforts in those countries. However, the RCS evaluation highlighted the need to support researchers at different career stages.

**Table 1:** HERA capacity strengthening activities by level

Individual	Attendance at international research methods short courses and conferences Attendance at in-country courses and conferences Participation in online courses and masterclasses Supervision of PhD students Mentoring of LMIC and HIC researchers
Individual country team	In-country training delivered by senior HERA researchers Training sessions delivered by LMIC non-HERA activists and researchers Mentoring and training to all teams in research methods and theory by both HERA and external senior researchers from both HIC and LMIC Presenting and training students, researchers or professionals in LMICs Engaging LMIC stakeholders for example, government officials and non-governmental organisations Disseminating research findings through media Collaborating with other global health research groups English classes
HERA group	Annual international group workshop week: methodological training sessions and research presentations from LMIC country research groups Regular ECR-led ECR virtual peer-support and education group Academic writing workshop, funded through an ECR’s successful application for external funding Collaboration with another global health research group

ECR, early career researcher; HERA, Healthcare Responding to Violence and Abuse; HIC, high-income country; LMIC, low- and middle-income country.

In this paper, we use the World Bank’s classification^6^ of HIC and LMICs, as our funder used the same, thus it shaped the partnership. However, we understand that a more deliberate context description in research is recommended.^7^ LMIC in HERA were diverse: importantly, although we do not discuss their differences in depth here, each faced specific challenges that affected RCS, with issues of political and economic instability, freedom of movement and access to resources varying across participant countries. We would like to briefly discuss the challenges faced by the Palestinian team to illustrate this point. Working with RCS in VAW in this setting requires acknowledging the effects of armed conflict on women’s health^31^ and the need for a feminist analysis capable of interrogating colonial violence, a theme that is often overlooked.^23 32 33^ The HERA Palestinian team faced mobility restrictions, both within their own territory and when attempting to leave the country for international engagements, which impacted their ability to work and collaborate. Although these restrictions were already burdensome and considered unlawful under international law, the situation escalated in October 2023. Attacks on health infrastructure by Israeli forces compounded the health crises.^34 35^ In this context, HERA researchers in Palestine have faced extreme working conditions, such as hospitals invasions, gas bombings, witnessing the arrest of patients and dealing with the added stress of caring for individuals without identification, who are then buried anonymously. Such conditions have been shown to lead to high levels of stress, burnout and trauma^35^ which is critical to address in any realistic discussion of RCS.

Conversations about power dynamics in global health, mentioned earlier, shaped the RCS programme’s evolution. This included changes, for example, renaming ‘capacity building’ to ‘capacity strengthening’ (discussed later) and appointing joint training leads from two LMIC (initially there were only two leads, both HIC-based). This paper reflects both the outcomes of our discussions and our contribution to this ongoing conversation. It aims to share insights and reflections of the programme and examine how a programme embedded within a global health partnership navigated power imbalances, and lessons learnt.

## Methods

### Study design

The current article draws on interview data. NT, ER, CH and LJB developed the interview schedule, drawing on RCS literature and findings from the wider, ongoing mixed-methods RCS evaluation. The schedule explored perceptions and experience of RCS; programme activities; mentoring and supervisory relationships; impact of RCS within participants’ country; global health research infrastructure and governance; research outputs; leadership and teamwork; programme management; group-defined values; and programme sustainability. We made adaptations as pertinent topics arose during initial interviews, including the impact of the COVID-19 pandemic.

### Data collection

Interviews were conducted via videocall (March to July 2020) by NT and ER, ECRs external to HERA’s research group. CH emailed participant information and a consent form to all 35 HERA group members. NT or ER contacted interested parties to arrange an interview. Participants could be interviewed in English or with a translator. However, all chose English. Consent was recorded verbally before the interview. Participants were reminded they could decline to answer any question or stop the interview at any point. Demographic data was collected. Interviews were digitally recorded, professionally transcribed, then cleaned and anonymised by the research team. Raw and identifiable data were stored on a secure University of Bristol computer server.

### Analysis

NVivo 12 facilitated data analysis. The analysis, a collaborative effort by CH, ER and NT, employed a reflexive thematic approach to identify and report patterns.^36 37^ In the first phase, each researcher independently read three interviews, annotating transcripts in Word before convening to discuss and agree a preliminary coding framework. Inductive (data-driven) and deductive (pre-existing areas of interest) analyses generated themes. The remaining transcripts were coded using the framework which was modified during analysis meetings. Joint analysis meetings between CH, ER and NT enabled them to engage reflexively on their own perspectives and potential biases. They discussed themes, and defined and refined them as more data was analysed. LJB viewed anonymised extracts (themes, subcategories and related quotes) to enable supervision of the analytical process, but did not analyse raw data. Conference and meeting presentations further aided in theme development, review, refinement and naming. The anonymised thematic framework was shared with AFd’O, who contributed to data interpretation. This step was significant as it prompted UK coauthors to critically re-examine interpretations and assumptions from a non-Eurocentric perspective.

### Reflexivity

Since our paper addresses power dynamics, author positionality and its influence on the paper, is relevant. NT, ER, CH and LJB designed and implemented the RCS programme evaluation. NT and ER were external to HERA, intended to enhance participants’ comfort and ensure anonymity. NT was a pre-doctoral fellow and ER a Masters in Public Health student. Both are native English speakers. Most authors (NT, ER, CH, SD, LJB) were HIC-based: authorship represents three HERA LMICs. Resultantly, the paper may predominantly reflect these country teams’ perspectives. Our plan for a third LMIC ECR coauthor (from oPt) were initially prevented by competing demands of their clinical work and the war. LMIC authors (BK, AFd’O) are affiliated with an academic institution with stable funding independent of HERA in a country without major political or economic instability. Initially, NT was the sole first author: we decided to include a joint-first LMIC author (BK) given her current PhD research on power dynamics in global health partnership, work on the decolonisation of the VAWG field, and experience as HERA RCS programme joint-training lead.

BK and SD were research participants. They did not contribute to the initial analysis, but their insights have influenced the paper. Despite the potential for bias, their perspectives enhance the credibility of the findings.^28^ As a researcher from an LMIC, BK’s and AFd'O worldview and assumptions provide valuable context, depth and a more critical perspective on power dynamics in global health partnerships. The topic landscape has evolved since the interviews, prompting us to present nuanced participant experiences without drawing definitive conclusions about RCS.

As we hope this reflexivity section makes clear, the issues described in this paper as influencing our RCS programme also shaped the paper itself. All coauthors are cisgender women with qualitative experience. Three held PhDs, three held Masters and two were clinical doctors.

### Patient and public involvement

Patients and/or the public were not involved in the design, or conduct, or reporting, or dissemination plans of this research.

## Results

18 HERA researchers participated (demographics in table 2). No ECRs, but most mid and senior researchers, were UK-based. Participants clearly articulated issues of power, often linking them to elements of RCS, which they primarily viewed as a way to address these challenges. Additionally, they expressed diverse opinions about RCS—generally positive—alongside critical perspectives on global health as a field characterised by inequalities. We categorised our findings under two themes: *Diverse understandings of RCS:* (1) mutual learning and (2) personal and team career development; and *Multiple hierarchies influencing power dynamics*, in (1) global health research environment, (2) HERA group and (3) individual country teams.

**Table 2:** Sociodemographic characteristics

Demographic	Participants, n=18
Geographic location*	
Brazil	2
Nepal	3
Occupied Palestinian territories	2
Sri Lanka	4
UK	7
Research career level	
Early	9
Mid	4
Senior	4
Other	1
Gender	
Female	15
Male	3
Age	
25–29	6
30–39	5
40–49	3
50+	4

* Nation of residence and employment.

### Diverse understandings of RCS

Participants articulated two aspects of RCS that afforded opportunities: (1) mutual learning, understanding and collaboration and (2) personal and team career development.

#### Mutual learning

UK participants expressed the importance of reciprocal learning between all partners to challenge the colonial model where UK partners are positioned as ‘experts’ and others as ‘learners’. One UK participant described an RCS qualitative methodology session, which used LMIC country partners’ data, as an opportunity for bidirectional learning. Session attendees, who had conducted the interviews, provided valuable depth and insight into the country context for UK session leaders, while also developing their skills in qualitative analysis.

[RCS] is a way of trying to elicit reciprocity: in return for [conducting research with] these populations, we’re giving something in return which is research skills. (P1-UK)

One UK participant reflected on the gradual realisation, notably after spending time as a group face-to-face, that there was much for everyone to learn from HERA’s partnership; ‘we are assuming that they … have to learn from us, and we have very little to learn from them, which is not very true …. I definitely think that the realisation that we’re going about this the wrong way was after [the first workshop week]’. Growing awareness (and discomfort) within the group of the negative connotations associated with the term ‘capacity building’ resulted in a consensus to use ‘capacity strengthening’ instead, a term suggested by oPt-based researcher. One of the ECRs found this shift positive and respectful of LMIC partners’ existing capacity and knowledge.

#### Personal and team career development

ECRs described RCS as a way of sharing knowledge, improving research skills and developing their research practice experientially as they engaged in different stages of their country specific research studies/PhDs. There was a sense of space and encouragement in which ECRs could engage with the HERA partnership, emerging from a recognition of the imbalance of power between countries.

HERA has this policy … to make this imbalance [on colonialism and research (terms the participant used earlier)] a bit easier … they have a lot of spaces for low-and-middle-income countries to do the presentations and to exchange knowledge and I actually think [that is what] capacity strengthening is all about. (P13-Brazil)

### Multiple hierarchies influencing power Dynamics

This theme is concerned with power dynamics within the RCS programme across three levels: the global health research environment, the HERA partnership and individual country teams. Participants often spoke of power implicitly that is, without using explicit terms like power or colonialism, conveying their experiences of power through experiences with communication, funding, publishing or hierarchy.

#### Global health research environment

The wider global health research environment—including challenges related to publishing, language barriers, applying theory and funding allocation—impacted HERA researchers. These systemic issues influenced how knowledge was produced and disseminated, often favouring institutions and individuals from HIC. Participants from Brazil, oPt, Sri Lanka and Nepal identified specific barriers to publishing in international journals. The publishing environment was contradictory: LMIC researchers were encouraged to publish LMIC produced knowledge of benefit to the LMIC context in ‘international’ journals that preferred, and were biased towards, the English language and HIC first authors. Moreover, according to them, when published in international journals this knowledge largely remained out of reach for LMIC individuals, who could benefit from it, for example, (in the HERA context) healthcare providers, clinic managers and policymakers.

We do have experiences of receiving ‘no’s’ when we’re first authors in international journals, and we don’t have [any]one from the United States or Europe in the authorship. I also think that we have a very specific construction of theory about health and about gender-based violence. Our PIs and co-PIs have been studying this since the 90s and they were the first group to start this in Brazil. So, they have this big journey in lots of theories about gender-based violence in a primary healthcare setting and the health sector in general and our theories are less valued [than] theories from high-income countries, it’s collective health. (P13-Brazil)

There’s this issue of national and international academic publishing in the sense that international academic publishing is more valued than national but international basically means Anglo-Saxon academic publishing. This is what international means. So, sometimes I feel that, not the HERA project, but the university and research structures, they incentivise us to publish outside [Brazil] when the knowledge that we’re producing would be very helpful here. (P2-Brazil)

Data LMIC partners collected were largely qualitative. Team analysis with UK researchers and PhD supervisors, or coauthoring articles for international journals, required language translation. LMIC research teams faced a dilemma about whether to analyse data in the original language or in English, with different country teams adopting varying approaches. Working with UK researchers and supervisors on translated data sometimes led to different interpretations between HIC and LMIC teams. Alternative interpretations were generally welcomed, but did not always match the experience in each country context.

One LMIC ECR reflected on the way power imbalances shape the theory used to inform findings and frame and articulate reality in academic publications. Theory-use was another way existing power dynamics between researchers from HICs and LMICs were reinforced. LMIC researchers’ perspectives and theoretical approaches were marginalised and underrepresented, thus perpetuating a knowledge hierarchy where some theories are more highly valued. It was also thought that drawing on these theories would enhance the likelihood of publication in prestigious international journals.

I think to publish internationally we have to use Anglo-Saxon theory, we’re not able to use a theory … that’s useful to understand our healthcare system, and of course, internationally you need theories that everyone can use … so you can have a discussion but … the issue is that those theories always come from the same places. So, publishing means adjusting your thoughts to the thinking of those places. (P2-Brazil)

The recognition that theories can be developed within LMIC contexts was highlighted by this LMIC researcher. They recollected an annual HERA group meeting where two LMIC PIs from different countries led a workshop on feminist theory and its origins and relevance to VAW in their contexts.

Participants recognised the need for LMIC researchers to seek funding from HIC donors due to funding shortages in their respective countries. The process of funding distribution also perpetuated global health research inequity and implied mistrust in the grant-management capacity of LMIC academic institutions.

I [see] tensions around how funding is allocated and I see it as a perfect example of neo-colonialism approach when a big rich wealthy country decides to give some money to a project, but they want this money to be distributed via a [UK] based university, which to me indicates lack of trust or … colonial approach … to me it’s a perfect illustration of a power imbalance. (P6-UK)

#### HERA group level

Two key elements of HERA’s RCS programme were perceived to mitigate power imbalances: mutual learning and the team’s informal, friendly approach. This supportive environment, especially valued by ECRs, fostered research collaboration and strengthened RCS. It promoted a sense of group connectedness, with open relationships encouraging LMIC researchers and ECRs to freely share opinions, influenced by the research leaders’ open communication and responsiveness.

You never feel this hierarchy thing which coming from a South Asian country you have it so much, whereas when you’re working with the team you don’t feel it at all which is amazing. (P5-Sri Lanka)

Relationship building, crucial for the RCS programme, was strengthened during in-person workshop weeks, with the second annual workshop fostering more collaboration and methodological training led by LMIC researchers and across institutions. However, participants noted that non-fluent English speakers faced significant barriers in fully benefiting from RCS activities, as English dominates global health research. This language barrier impacted their ability to communicate, share ideas, network, participate in workshops and publish. Participants suggested acknowledging and addressing this issue through open team communication to identify mitigation strategies, such as having English classes as part of RCS.

Researchers based outside of the UK referred to resources, training and networks they accessed through HERA’s RCS programme and how these provided career advancement opportunities unavailable in their own country. They felt being HIC institute-affiliated increased the prestige that others associated with their work; ‘there is this trust factor … these universities are renowned, and they have a … good reputation … especially in a country context like ours where hierarchy plays such an important role’ (P7-Nepal).

Participants believed their input in shaping RCS activities and goals, such as these training opportunities, promoted equity within the group. Early and mid-career researchers highlighted that training opportunities were tailored to collective suggestions. Beyond training, views on the value of degrees from HIC universities varied among non-UK participants; while one valued pursuing a home-university PhD for its relevance, another viewed a UK PhD as more prestigious, citing a lack of expertise in their countries and appreciating the UK’s non-hierarchical teaching/supervision style. Participants also mentioned HERA-funded PhD students had better access to study funding and opportunities than the home-university PhD due to difference in the funder rules.

Because we don’t have any ECRs [early-career researchers] funded by the group, we don’t have the same opportunities but I don’t think …this is something that has to do with the HERA team, it has to do with the funder. (P2-Brazil)

Linking to P6’s comment in the previous theme, the HERA partnership acknowledged the unstable economic situations of LMIC researchers by adapting funding disbursement methods. A UK-based PI appealed for and secured advance payments to partners and PhD students instead of reimbursements, benefiting those directly involved *and* another UK institution in the collaboration.

#### Individual country teams

Hierarchy in teams had varying impact on RCS: power dynamics differed across countries. In some teams, hierarchical structures hindered RCS more than in others, influenced by each team’s academic environment and broader professional culture.

Participants observed that team hierarchies in other countries stemmed from experience and subject knowledge. Hierarchy was not seen as authoritative or an impediment, but a structure to support others in varying career stages. Ostensibly hierarchy was associated with expertise in the field and leadership skills rather than a source of inequity or division.

I really learned a lot from the team and the way they … lead their research team … they have weekly meetings with their junior staff researchers where they discuss issues, concerns and roles of each of the researchers and it’s done in a very participatory way … It’s hierarchical but it’s also informal. (P3-Sri Lanka)

## Discussion

Participants clearly recognised the power inequalities between HICs and LMICs and acknowledged the need to address them. This recognition translated into ideas for RCS and adaptations to the RCS programme and the whole project. Issues of economic, epistemic and normative power imbalances were also mentioned, often alongside potential mitigation strategies.

Participants valued ideas of reciprocity and bidirectionality as ways for RCS to address inequalities, with mutual learning identified as a core value of the RCS programme. However, those views were sometimes expressed in ways that did not necessarily challenge epistemic power imbalances. For example, it was suggested that LMIC researchers could contribute insights into what is ‘local’, such as language and context, while HIC would provide theoretical frameworks through methodological knowledge. Over time, interactions between HIC and LMIC researchers seemed to shift this perception, fostering a more authentic experience of mutual learning. Notably, as mentioned in the interviews, LMIC researchers conducted workshops focused on feminist theory, its origins and relevance to VAW in their specific contexts. These presentations were especially important considering the centrality of contextual awareness in VAW research to ensure its safety, effectiveness and relevance, and the critiques of the predominance feminist ideas and methodologies originating from HIC contexts in the VAW field.

The RCS programme included an English academic writing workshop organised by LMIC ECR who recognised the importance of publishing in English in international journals for advancing their careers. However, interviews revealed that incentives in this direction, coming from the broader academic environment, could undermine the RCS ability of creating of social value. Some LMIC researchers noted that incentives to publish in these journals often compelled them to adopt HIC-centric approaches and theoretical frameworks instead of those created in and used for their context. They perceived the push for international publications as precluding accessibility of knowledge produced for key LMIC stakeholders due to language barriers and restricted journal access. This finding illustrates Abimbola’s^20^ argument that the intended audience and choice of publication are critical aspects of publishing. It also exemplifies the challenges RCS programmes face in advancing LMIC researchers’ careers within an environment where excellence is defined by HIC standards.^5^ Additionally, it highlights the difficulties of challenging broader structures of normative and epistemic power at the level of a single project, as many participants perceived it as difficult to publish in these journals with LMIC first authorship or without an HIC partner.

Affiliation with, and PhDs from, UK universities were sometimes considered advantageous due to perceived expertise, non-hierarchical teaching/supervision styles, and prestige. This perception again highlights the challenge RCS programmes face in balancing broader issues of epistemic and normative power in global health and academia with the valid desire of ECR for career progression in a field dominated by HIC institutions. Additionally, English language dominance reflects issues of power in global health, as researchers’ proficiency affected access to the benefits of certain RCS activities, their integration within the group, and opportunities for career advancement. The RCS programme incorporated English classes at the request of participating countries for this reason. This solution may help mitigate inequalities at group level but does not address the broader structural issues.

Economic power was also seen as perpetuating inequalities, including the colonial legacy of funding structures. Changing the way funding was disbursed could be seen as strengthening UK universities’ capacity to conduct global health research. While this kind of change alone is insufficient to address broader economic imbalances, it helped to manage the imbalances within this particular partnership.

Participants noted that informal relationships within HERA played a key role in mitigating power imbalances by diminishing perceived hierarchies. The RCS programme facilitated these connections through annual workshops and ECR-led online meetings. It is important to note that access to those opportunities were unequally distributed: internet connection, political and economic stability and the varying levels of freedom that passport holders of different nationality enjoyed affected access. Although funding for HERA has ended, researchers within the partnership continue to engage in RCS activities, including LMIC and HIC research impact events, conferences, and joint paper-writing, ensuring that the collaborative spirit and capacity-strengthening efforts the programme initiated remain active and impactful.

HERA’s RCS programme evolved over time to address power imbalances stemming from the broader global health environment, incorporating solutions and learning opportunities along the way. This evolution was reflected in the name ‘capacity strengthening’, acknowledging existing capacity and expertise in LMIC. Participant input in shaping the agenda, and the addition of RCS co-leads from two LMIC, further reflected the need for increased LMIC leadership.

Our study shows that meaningful change in RCS depends on transformations in the broader global health and academic environment. Inequalities in partnerships and in their RCS efforts reflect issues such as HIC dominance in funding and its consequences for agenda-setting and power distribution within partnerships. When combined with international publishing as a metric of success in an English language dominated publishing environment, this furthers the tendency to have HICs’ theories and understanding universalised. Addressing those issues is important not only to guarantee that RCS can move forward, but also to remove barriers this environment creates for LMIC researchers to have the space and resources to conceptualise their own realities and imagine better solutions to their problems.

### Strengths and limitations

This study provides a unique opportunity for transformation through learning from the challenges faced by an RCS programme concerned with addressing power dynamics and to generate a deep understanding of experiences of RCS activities, particularly in relation to global health power asymmetries within an international research partnership focused on VAW. This multi-country research study was conducted in four countries outside the African continent, which has produced most of the existing evidence on RCS. Social desirability bias may have shaped responses and conducting the interviews in English may have limited participation. Data collection was part of a broader evaluation of the HERA RCS. While we prioritised anonymity, this may have affected participation and responses provided.

## Conclusions

Despite a strong effort, progress was often hindered by the fact that these inequalities were rooted in broader structural issues in global health research, as seen within HERA. The programme also faced the challenge that strategies to advance researchers’ careers and build capacity under the current model sometimes conflicted with creating social value or challenging epistemic and normative structures. Participants clearly recognised power imbalances within the partnership and were keen to address them through the RCS programme. This led to a steep learning curve and significant adaptations to make progress within existing structural limitations.

## Data Availability

No data are available.
